# Epidemiology of Chronic Shoulder Pain Among Adult Patients in a Tertiary Care Hospital in South India

**DOI:** 10.7759/cureus.67982

**Published:** 2024-08-27

**Authors:** Rinju Krishnan, Mahesh Shekoba, Farah N Fathima, Mevin M Nedumparampil, Anoop Pilar, Shreeraam Venkatachalam, Rajkumar Amravathi

**Affiliations:** 1 Orthopaedics, St John's Medical College, Bangalore, IND; 2 Community Medicine, St John's Medical College, Bangalore, IND; 3 Orthopaedics, Kurinji Superspeciality Hospital, Salem, IND

**Keywords:** epidemiological study, frozen shoulder, rotator cuff pathology, repetitive movements, chronic shoulder pain

## Abstract

Introduction

Chronic shoulder pain (CSP) is a significant medical and socioeconomic problem that hinders daily living activities, creating a burden on the individual and society. An epidemiological study will help to find out the risk factors and their impact, thereby giving medical practitioners and policymakers the necessary tools to tackle the problem.

Materials and methods

This is a cross-sectional study conducted in a tertiary care hospital in South India over a period of four years from 2018 to 2021 using a structured questionnaire, clinical and radiological examination, and visual analog score (VAS). The data were analyzed using SPSS software.

Results

A statistically significant correlation was found between CSP and increasing age, occupational risk factors like vibrations, repetitive movements, lifting heavy objects, repetitive lifting of the arm above the shoulder and sitting in the same posture for a prolonged time, and work stress. Vitamin D deficiency and diabetes mellitus were found to increase the risk of shoulder pain, smoking, and alcoholism. There was a higher incidence of rotator cuff pathology and frozen shoulder among those who had CSP.

Conclusion

CSP affects the quality of life and the productivity of the patients. Reducing the physical and psychosocial risk factors is the key to decreasing its prevalence. Maintaining a healthy lifestyle and a good working environment is very essential.

## Introduction

Shoulder pain is common among the general population, and also a common presenting symptom to health care practitioners. There was no descriptive epidemiological study available regarding this among the South Indian population. The prevalence of chronic shoulder pain (CSP) varies greatly among the populations that have been investigated, and this variation may be caused by the many definitions of shoulder pain that have been proposed. The epidemiological study of the shoulder is reportedly plagued by four methodological issues, according to Bjelle: criteria and categorization, study design, diagnostic process, and techniques for evaluating risk factors [1]. Also, there might be variability in reporting, as the shoulder can be considered as a primary or secondary source of pain, making the clinicians and authors summarize the presentation either as a shoulder pain syndrome or just as a shoulder pain [1].

The risk factors for CSP can be categorized as personal risk factors and work-related physical and psychosocial factors. Personal risk factors include sleep disturbances, consumption of caffeine and alcohol, and smoking [2,3]. Work-related risk factors include work-related posture, repetitive work, repeated overhead activity, high force demand and vibration, and computer work [4]. The primary predictor of a patient's outcome when they see a healthcare professional for shoulder discomfort is the patient's initial visit pain level and any prior shoulder issues [5]. The epidemiological data from our study will help to identify the causes and sociodemographic profile of the patients who develop CSP in the south Indian population and could be extrapolated to the whole Indian population

## Materials and methods

This is a cross-sectional study carried out in a tertiary care hospital in south India for a period of four years from 2018 to 2021. The subjects of the study were patients above 18 years who presented to the outpatient with complaints of shoulder pain for more than 90 days. Patients with fractures around the shoulder, tumors, infections, referred pain, and concomitant cervical spine and shoulder pathology were excluded. The sample size of the study was 196 patients. For analysis, the patients were grouped into age groups (<30, 31-40, 41-50, 51-60, and >60). Our institution caters to all strata of people in about 10 districts of three states, of both rural and urban areas in south India. The objective of the study is to create a sociodemographic profile of patients with CSP among the South Indian population and to study the risk factors leading to CSP and its socioeconomic implications. Standard shoulder radiographs (anteroposterior view, lateral view, and scapular Y view) and if needed magnetic resonance imaging (MRI) were taken to evaluate CSP. Data was collected using a structured questionnaire, visual analog score (VAS), and thorough clinical examination. Data were analyzed using SPSS software. Association was tested using the chi-square test, and a p-value of <0.05 was considered significant. An independent sample t-test was done with the VAS score as a dependent variable and the different sociodemographic and disease-related risk factors as predictors. Multiple linear regression was done with the VAS score as a dependent variable and the different sociodemographic and disease-related risk factors that were associated with the severity of pain by variate analysis as predictors. An ANOVA test was also performed to ascertain the severity of particular shoulder diseases.

Questionnaire

A self-administered questionnaire was developed for the study (Table 6 of Appendix). It had four parts. The first part included questions on the general sociodemographic profile of the patients like age, sex, educational status, marital status, family income, type of family and number of family members, dominant hand, smoking, and alcoholism. The second part includes details of the patient’s occupation, which includes working hours, ergonomic factors associated with the work inspired by the Dutch Musculoskeletal Questionnaire, like Lifting heavy weights, exposure to vibrations, lifting the arm above the shoulder level, using wrist and arm at a repetitive way, sitting/standing for long periods of time, and whether it is a physically demanding job, and questions relating to psychosocial risk factors, like stress at work, job satisfaction, support and help from co-workers and superiors, and leave application in the past three months. Part three includes details of the CSP like site of pain, severity of pain by VAS, duration of pain, duration of sleep, details of previous consultations, and details of co-morbidities. Part four is a physical examination including height (cm) and weight (Kg), range of movement, special test for rotator cuff, impingement, labral tear, and biceps tendinitis.

## Results

Demographics

Out of the 196 study subjects, 123 (62.8%) were males and 73 (37.2%) were females. A total of 62% of the people were above the age of 40 years (Table 1).

**Table 1:** Gender distribution among various age groups N, number of patients

Age		Gender		Total
		Male	Female	
	<30 years	31	3	34 (17.3%)
	31-40 years	29	11	40 (20.4%)
	41-50 years	17	22	39 (19.9%)
	51-60 years	30	22	52 (26.5%)
	>60 years	16	15	31 (15.8%)
Total		123 (62.8%)	73 (37.2%)	196 (100%)

Among the subjects, 44.4% were graduates, 27.5% were 12th pass, and 28% were uneducated. The majority of the patients, about 69.4%, were married and about 61.2% belonged to a non-nuclear family. The mean number of family members was around 4.29+2.77 while the mean working hours per day was around 5.02+3.86. About 84.2% of the patients who presented with CSP were right-hand dominant. A statistically significant correlation was obtained between the side of shoulder pain and the dominant hand (chi-square: 9.183, p<0.05). The mean duration of pain is around 127.69 days. The intensity of the pain was analyzed using VAS. The majority of the patients (62.8%) had moderate pain with VAS between 4 and 7. However, the mean duration of night sleep was around 8.27+2.27 hours. About 67.3% of patients had already consulted a doctor before coming to our tertiary care center, while about 32.7% of patients had sought alternative systems of treatment.

Risk factors

On analyzing the occupation among the study subjects, CSP was found to be more common among housewives and the other group, about 30.1% each, while among information technology (IT) professionals it was around 18.4% (Table 2). 

**Table 2:** Distribution based on various occupations IT, information technology

Occupation	Frequency (N)	Percent (%)
IT sector	36	18.4
Sportsmen	9	4.6%
Manual laborers	22	11.2%
Housewife	59	30.1%
Unemployed	11	5.6%
Others	59	30.1%
Total	196	100.0%

There was a statistically significant association between occupational risk factors and shoulder pain (chi-square: 49.765, p<0.05). Considering the risk factors involved during work it was found that about 17.3% were exposed to vibration, 60% of the patients had a history of heavy weight lifting, 70.4% had a history of sitting in the same posture for a prolonged time, 78.6% of patients had a history of lifting the arm above the shoulder while working, 84.2% had a history of using the arm in a repetitive way, and 88.8% feel that their work is physically demanding. On multiple linear regression, lifting heavy weights emerged as the only predictor of shoulder pain (b-coefficient: 0.13 and p-value: 0.05). There was a strong association between using the arm in a repetitive way and CSP (chi-square: 11.587, p<0.05). While among the study subjects, 96% with CSP feel stressed at work and are unable to carry out work normally (chi-square: 11.622, p<0.05). 82.7% of patients feel shoulder pain in some way or other affects their work. About 58.2% of patients have applied for leave in the past three months due to shoulder pain affecting their income and productivity, which was found to be statistically significant (chi-square: 20.165, p<0.05) (Table 3).

**Table 3:** Sociodemographic and disease-related predictors of severity of CSP *P-value to be significant if <0.05. N, number of patients; CSP, chronic shoulder pain

Variable	Categories	N	VAS score	P-value
Age	<50/ >50	108/88	4.7±1.1/5.0±1.2	0.02*
Gender	M/F	123/73	4.8±1.2/4.8±1.0	0.587
Dominant hand	Right/left	165/31	4.8±1.1/4.9±1.0	0.47
Lifting weight	Yes/no	118/78	5.0±1.1/4.5±1.2	0.01*
Lifting arm above shoulder	Yes/no	154/42	4.8±1.1/4.6±1.3	0.18
Exposure to vibration	Yes/no	34/162	4.8±1.1/4.8±1.1	0.96
Using arm in repetitive way	Yes/no	165/31	4.8±1.1/4.9±1.1	0.58
long time in same posture	Yes/no	138/58	4.7±1.2/5.1±1.0	0.02*
Physically demanding	Yes/no	174/22	4.8±1.1/5.0±1.1	0.26
Gout	Yes/no	21/175	5.0±1.3/4.8±1.1	0.36
Rheumatoid arthritis	Yes/no	22/174	4.9±0.8/4.8±1.2	0.74
Osteoporosis	Yes/no	46/150	1.0±1.3/4.7±1.1	0.20
Vitamin D deficiency	Yes/no	125/71	4.9±1.1/4.5±1.1	0.02*
Diabetes	Yes/no	76/120	5.0±1.2/4.7±1.0	0.08
Working hours	0-8/>9	155/41	4.8±1.1/4.8±1.1	0.89

In our study, we looked into the association of CSP with various medical conditions and we found the strongest association with vitamin D deficiency in about 63.8% of patients (chi-square: 13.425, p<0.05), while only 38.8% of patients had diabetes mellitus. Also, vitamin D deficiency turned out to be a major predictor of CSP (p<0.05) when compared to other risk factors when multiple linear regression was done with VAS score as a dependent variable.

About 23.5% of patients had osteoporosis, 11.2% of patients had rheumatoid arthritis, and 10.7% had gout. In our study, there were about 64 (32.7%) smokers and 83 (42.3%) alcoholics. In our study, a strong association between CSP and high body mass index (BMI) was found; there were about 132 (67%) overweight patients with a BMI of 25-29.9 and eight (4.1%) patients with a BMI above 30.

Underlying pathology

On evaluating the underlying pathology, it was found that about 73 (37.2%) patients had cuff pathology, 42 (21.4%) had impingement, 35 (17.9%) had frozen shoulder, 33 (16.8%) patients had a labral tear, eight (4.1%) had subacromial bursitis, and 5 (2.6%) had bicipital tendinitis. The labral tear was more common in the younger age group (<30 years), while shoulder impingement was more common in the age group of 31-40 years, and frozen shoulder and rotator cuff tears were more common after 40 years (Table 4).

**Table 4:** Comparison of age categories with the diagnosis N, number of patients

Age(years)			Diagnosis						Total (N/%)
			Cuff pathology (N/%)	Frozen shoulder (N/%)	Shoulder impingement syndrome (N/%)	Labrum tear (N/%)	Biceps tendinitis (N/%)	Subacromial bursitis (N/%)	
	<30		3	0	11	19	0	1	34
			(8.8%)		(32.4%)	(55.9%)		(2.9%)	(17.3%)
	31-40		12	3	13	6	3	3	40
			(30.0%)	(7.5%)	(32.5%)	(15%)	(7.5%)	(7.5%)	(20.4%)
	41-50		18	10	5	4	0	2	39
			(46.2%)	(25.6%)	(12.8%)	(10.2%)		(5.1%)	(19.8%)
	51-60		28	14	7	3	0	0	52
			(53.8%)	(26.9%)	(13.4%)	(5.7%)			(26.5%)
	>60 years		12	8	6	1	2	2	31
			(38.7%)	(25.8%)	(19.3%)	(3.2%)	(6.4%)	(6.4%)	(15.8%)
Total (N/%)			73	35	42	33	5	8	196
			(37.2%)	(17.8%)	(21.4%)	(16.8%)	(2.5%)	(4.1%)	

Except for frozen shoulder, all other pathologies were more common in males. On comparing these pathologies with the educational status of the patient, it was found that frozen shoulder (42.9%), cuff tears (39.7%), and subacromial bursitis (62.5%) were more common in uneducated patients, while shoulder impingement (54.8%) and labral tear (75.8%) were more common in graduates. In comparison to other shoulder diseases, cuff tears and labral tears were shown to have higher mean VAS scores (mean>5) in the ANOVA test used to measure severity (Table 5).

**Table 5:** Distribution of severity of CSP among shoulder-specific pathologies *Shoulder pathologies with higher mean VAS scores (mean >5) in the ANOVA test. N, number of patients; CSP, chronic shoulder pain; VAS, visual analog score

Shoulder pathologies	N	Mean	SD	Test statistics and P-value
Cuff pathology*	73 (37.2%)	5.37	1.048	F=12.064, P<0.001, df=5
Frozen shoulder	35 (17.8%)	4.86	0.974	
Impingement syndrome	42 (21.4%)	3.90	0.958	
Labral tear*	33 (16.8%)	5.03	1.132	
Biceps tendinitis	5 (2.5%)	4.40	1.342	
Subacromial bursitis	8 (4.1%)	4.13	0.641	
Total	196	4.83	1.162	

## Discussion

According to some authors, the total prevalence of shoulder pain in the UK population is believed to be 7%, but in the elderly, the statistic rises to 26% [6,7]. In a population-based study in Norway, Hasvold et al., according to estimates, the prevalence of shoulder pain was 24.9% in women and 15.4% in males [8]. In a research published in 2005 by Bot et al., women had more consultations for shoulder complaints (31.4 per 1000) than men (23.2) [9]. In our study, shoulder pain was predominant in males (62.8%) compared to females (37.2%). This is contrary to the general population, which could be because males with shoulder pain are the ones who visit tertiary care for treatment more than females. Our observations were similar to the study by Van der Windt et al., where the prevalence was 54% in men and 46% in females [10]. In our study, female predominance was seen in the age group 41-50 years, and after 60 years, equal distribution was seen among both sexes. There are several theories that could explain why shoulder complaints would be more common in women. One of these is that women's employment typically includes repetitive, low-control tasks that put static stress on the neck and shoulder muscles more regularly than those of males [11]. Also, in women, there is a tendency to label stimuli as more noxious and have lower pain thresholds [12].

The prevalence of shoulder pain is observed to increase with age in our study. This is similar to research by Bot et al. that gathered information from 195 general practitioners located throughout the Netherlands [9]. The age groups of 40-49 and 50-59 saw the highest prevalence, with a greater proportion of females than males. In our study, the age group of 51-60 years had the highest prevalence. Hence, it can be inferred that as age progresses, the degenerative changes increase, leading to a higher prevalence in the older age group. In a study by Vikat et al., it was discovered that girls aged 16 to 18 frequently experienced shoulder pain, which was strongly correlated with psychosomatic symptoms [13]. Another finding in our study was the statistically significant relation between CSP and the dominant hand, implying more wear and tear in the overused hand.

Most of the patients who visited us were graduates (44.4%), while those educated till 12th standard were the least (27.5%). This result was comparable to that of the Per-Olof Ostergen research in which graduates made up 30% of the patient population [14]. On the other hand, studies based on populations differ from this. Therefore, it stands to reason that better-educated patients seek medical attention since they are more cognizant of and aware of health issues. Compared to nuclear families, non-nuclear families with low yearly incomes had a higher prevalence of CSP. Due to the longer workdays, having several family members is a risk factor for shoulder pain. 69.4% of the patients in our study were married, which was similar to Per-Olof Ostergren's study, which found 60% of the participants to be married. This demonstrates the correlation between persistent shoulder pain and a variety of socioeconomic characteristics [14].

Occupation is an important determining factor in CSP. Concerning our study, pain was found to be more common among housewives and the other group, 30.1% each, followed by IT professionals (18%). The increasing prevalence of pain among IT professionals was also seen in a study conducted by Eltayeb et al. [15]. It is expected that a majority of working individuals in Western societies presently utilize personal computers for work purposes. Psychosocial factors such as time constraints and high quantitative demands at work, together with prolonged and sustained postures, continuous force, and excessively repetitive motions, are all impacted by this [11]. This result is consistent with our study, which indicated that prolonged sitting was a major predictor of CSP (Table 4). Additionally, this has led to a rise in shoulder-related diseases, particularly in young people and adolescents [16,17]. This is made worse by teenagers' easy access to the internet, usage of cell phones, and computer games, all of which have created a new set of risk factors for shoulder pain. In an investigation of teenagers between the ages of 14 and 18, Hakala et al. found that daily computer use of more than two to three hours appears to be a threshold for neck or shoulder pain, which could account for the correlation between rising computer-related activities and rising shoulder-related pain in young people [18]. Incorporating work breaks, decreasing reflection or glare, and raising the screen are crucial elements to include in the design of computer workstations in the future.

A statistically significant correlation was seen between CSP and occupational risk factors like exposure to vibrations, repetitive movements, lifting heavy weights, lifting the arm above the shoulder, and sitting in the same posture for a prolonged period. Lifting heavy weights was the only variable that could predict shoulder pain in multiple linear regression (b-coefficient: 0.13 and P-value: 0.05). This is comparable to the study conducted by Van der Windt et al. [19]. Additionally, there is a clear link between the beginning of shoulder pain and repeated work [20]. Of the people we studied, 96% report feeling under stress at work. Employees with high exposure to both psychological and physical workplace risk factors were found to be more likely than those with high exposure to either risk factor to report symptoms of musculoskeletal diseases, including shoulder abnormalities. According to Devereux et al., employees who meet high psychosocial exposure criteria, such as having high mental demands, little job control, and little support from peers and society, are more likely to develop CSP [21]. One of the biggest risk factors for shoulder pain that started recently was monotonous employment. Moreover, shoulder pain is very common among athletes, particularly in overhead sports like baseball, tennis, swimming, and other activities where the shoulder must be used repeatedly. Engaging in overhead activities exposes the shoulder to several factors such as strain, exhaustion, microtrauma, laxity in the static stabilizers, and imbalances in the muscles of the dynamic stabilizers. These factors can lead to changes in the shoulder's mechanical function and increase the risk of injury.

In our study, more than half of the patients have applied for leave over the past three months, which is comparable to the study conducted by Bergenudd H et al., which showed that patients were more likely to take sick leave due to pain, affecting their income and productivity of the work [22]. It was discovered that those in the groups with moderate to high physical demands had a higher probability of experiencing pain and needing sick leave as a result of their impairment. Additionally, the main psychosocial element impacted by pain patients was low job satisfaction.

In our study, 67.3% of patients had already consulted a general practitioner before coming to the tertiary care center. 32.7% of patients had already sought an alternative system of treatment before coming to our center. These observations were comparable to the study conducted by Breivik H in a European-based survey of 15 countries, where one-third of the patients with CSP were currently not being treated and two-thirds sought care from the alternative system of treatment [23]. In our study, the majority of the patients had moderate pain scores (4-7) in VAS. This shows that patients with low VAS seek care from a general practitioner or an alternative medicine practitioner. Various co-morbidities are considered risk factors for shoulder pathology like gout, rheumatoid arthritis, osteoporosis, diabetes mellitus, and vitamin D deficiency. Vitamin D deficiency was found to have a significant association with CSP. In our study, 38% of patients were diabetic, which is comparable to the study conducted by Anna Grimby-Ekman [24].

Smoking and alcohol have an association with the development of shoulder pain. In our study, 32.7% of patients were smokers, and 42.3% consumed alcohol. This is comparable to the study conducted by Beifeng Chen, which showed 34% smokers and 36% alcoholics [25]. CSP is associated with various underlying pathology like impingement, labral tear, cuff pathology, and biceps tendinitis. In our study, cuff pathology was the most common (37.2%) finding; this was comparable to the study by Van der Windt et al. conducted in the Netherlands where it was 29% [10]. Cuff pathology and labral tear were found to have higher mean VAS scores (mean: >5) among other shoulder pathologies in our study. Biceps tendinitis was the least common pathology in our group. Degenerative pathologies like rotator cuff tears and frozen shoulder were more common in older age groups unlike labral tears, which were more common in younger age groups. This study's findings are limited by its cross-sectional design and the fact that it was conducted at a single tertiary care hospital with a sample of 196 patients, which may affect the generalizability to the broader population. The reliance on self-reported data introduces the possibility of recall bias, and the exclusion of certain patient groups might have led to an underestimation of CSP prevalence. Additionally, the absence of specific shoulder scoring systems limits the depth of analysis regarding the functional impact of shoulder conditions. Finally, unmeasured confounders, such as genetic factors or other lifestyle variables, could have influenced the results.

## Conclusions

CSP affects the quality of life and productivity of the patients. Reducing the physical and psychosocial risk factors for the CSP is the key to decreasing the prevalence in the community. The study shows that maintaining a healthy lifestyle with exercise and avoidance of alcohol and smoking is important for the prevention of CSP. Also creating a healthy working environment and good relations with the supervisors and colleagues is important. Maintaining a healthy work-life balance is also the key. Imparting awareness regarding seeking early medical care, especially for those with co-morbidities is crucial. This epidemiological data will help in targeting the high-risk groups, thereby helping to decrease the burden of the CSP at an individual level and the level of society.

## Appendices

**Table 6:** Questionnare VAS, visual analog scale

Patient number													Hospital number																											
SECTION 1: SOCIODEMOGRAPHIC PROFILE																																								
SI No			Variable			Coding																																		
1.1			Name																																					
1.2			Age																																					
1.3			Gender			0 Male																														1 Female				
1.6			Education			0 Uneducated					1 Primary School																		2 Middle School											
						3 High School				4 PUC									5 Degree/Diploma														6 Postgraduation							
1.7			Marital status			0 Married			1 Unmarried												2 Separated/Divorced																3 Widow(er)			
1.8			Family type			0 Non-nuclear														1 Nuclear																				
1.9			Total family income																																					
1.10			Number of family members																																					
1.11			Dominant hand						Right														Left																	
SECTION 2: DETAILS OF OCCUPATION																																								
2.1	Occupation								0 IT Sector									1 Sportsmen													2 Manual Laborers									
									3 Housewife									4 Unemployed													5 Others									
2.2	Duration in present occupation																																							
2.3	Number of working hours per day																																							
2.4	Does your work involve any of the following: 0, No; 1, Yes; 2, Not applicable								Lifting heavy weights																		Exposure to vibrations													
									Lifting arm above the shoulder level																		Using wrist and arm in a repetitive way													
									Sitting/standing for long periods of time in the same posture																		Physically demanding													
2.5	Do you feel stressed while working?								0 No																		1 Yes													
2.6	Are you satisfied with your work?								0 No											1 Yes												2 Not applicable								
2.7	Is your supervisor supportive?								0 No											1 Yes												2 Not applicable								
2.8	Are your co-workers helpful?								0 No											1 Yes												2 Not applicable								
2.9	Does your shoulder pain affect your usual work?								Quantitatively									Qualitatively												Both					Does not affect work					
2.10	Have you applied for sick leave in the past 3 months due to shoulder pain?								0 No									1 Yes														2 Not applicable								
SECTION 3: DETAILS OF SHOULDER PAIN																																								
3.1	Site of pain							Right shoulder														Left shoulder												Both shoulders						
3.2	Severity of current pain by VAS																																							
3.3	Average severity of pain over the last 30 days																																							
3.4	Duration of pain in days																																							
3.5	Have you seen a doctor before for shoulder pain							No																Yes																
3.6	Have you sought care from any alternate system for shoulder pain																																							
3.7	Do you have any of the following medical conditions? 0, No; 1, Yes							Gout																Pseudogout																
								Osteoporosis																Rheumatoid arthritis																
								Vitamin D deficiency																Diabetes																
3.8	Average duration of sleep at night																																							
3.9	Do you smoke?																No								Yes															
3.10	Do you consume alcohol																0 No								Yes															
SECTION 4: PHYSICAL EXAMINATION																																								
4.1		Height in cms																																						
4.2		Weight in kgs																																						
4.3		Inspection (No/Yes)			Abnormal attitude (details)							Scar/sinus				Swelling												Muscle wasting											Deformity	
4.4		Palpation (No/Yes)			Tenderness							Local rise of temperature																												
4.5		Range of movements (normal/restricted)			Flexion		Extension							Internal rotation												External rotation													Abduction	
					4.6		Special tests					Positive																			Negative														
Hawkins test																																								
Neers test																																								
Drop arm sign																																								
Belly press test																																								
Obriens test																																								
4.7		Diagnosis: 1. Cuff pathology/2. Frozen shoulder/3. Impingement syndrome/4. Labrum tear/ 5. Biceps tendinitis/6. Subacromial bursitis																																						
